# Copper Bracelets and Magnetic Wrist Straps for Rheumatoid Arthritis – Analgesic and Anti-Inflammatory Effects: A Randomised Double-Blind Placebo Controlled Crossover Trial

**DOI:** 10.1371/journal.pone.0071529

**Published:** 2013-09-16

**Authors:** Stewart J. Richmond, Shalmini Gunadasa, Martin Bland, Hugh MacPherson

**Affiliations:** 1 Department of Health Sciences, University of York, Heslington, York, United Kingdom; 2 Health Services Research Unit, University of Aberdeen, Aberdeen, United Kingdom; The James Cook University Hospital, United Kingdom

## Abstract

**Background:**

Folklore remedies for pain and inflammation in rheumatoid arthritis include the application of magnets and copper to the skin. Despite the popular use of devices containing magnets or copper for this purpose, little research has been conducted to evaluate the efficacy of such treatments.

**Objective:**

To investigate whether the practice of wearing magnetic wrists straps, or copper bracelets, offers any specific therapeutic benefit for patients with rheumatoid arthritis.

**Design:**

*Randomised double-blind placebo-controlled crossover trial*.

**Methods:**

70 patients, aged 33 to 79 years and predominantly female (n = 52), with painful rheumatoid arthritis were recruited from general practices within Yorkshire. Participants were randomly allocated to wear four devices in a different order. Devices tested were: a standard (1502 to 2365 gauss) magnetic wrist strap, a demagnetised (<20 gauss) wrist strap, an attenuated (250 to 350 gauss) magnetic wrist strap, and a copper bracelet. Devices were each worn for five weeks, with treatment phases being separated by one week wash-out periods. The primary outcome measured was pain using a 100 mm visual analogue scale. Secondary pain measures were the McGill Pain Questionnaire and tender joint count. Inflammation was assessed using C-reactive protein and plasma viscosity blood tests and by swollen joint count. Physical function was assessed using the Health Assessment Questionnaire (Disability Index). Disease activity and medication use was also measured.

**Results:**

65 participants provided complete self-report outcome data for all devices, four participants provided partial data. Analysis of treatment outcomes did not reveal any statistically significant differences (P>0.05) between the four devices in terms of their effects on pain, inflammation, physical function, disease activity, or medication use.

**Conclusions:**

Wearing a magnetic wrist strap or a copper bracelet did not appear to have any meaningful therapeutic effect, beyond that of a placebo, for alleviating symptoms and combating disease activity in rheumatoid arthritis.

**Trial Registration:**

Controlled-Trials.com ISRCTN51459023 ISRCTN51459023.

## Introduction

### Background

Belief in the healing power of magnets and the practice of wearing magnetic objects to alleviate symptoms of arthritis is a tradition that spans two millennia [1]–[2]. Rituals involving magnets featured heavily in the astonishing cures of Anton Mesmer during the 18^th^ century, which led directly to the development of the controlled clinical trial [3]. In 1830, discovery of copper in the blood fostered new beliefs concerning a causal link between copper deficiency and rheumatism [4]. This resulted in a new system of treatment, referred to as metallotherapy, involving the application of copper discs and other metal objects to the bodies of the afflicted. Yet, whilst early controlled clinical research revealed the effects of both Mesmerism and metallotherapy to be purely psychogenic [5]–[6], similar beliefs and practices still persist today. Magnet therapy, in particular, is arguably far more popular now than at any other time in history, with estimated annual worldwide sales exceeding one billion pounds [7]–[8]. Devices, such as bracelets and insoles, which incorporate either permanent magnets or copper, are widely promoted for relieving pain and combating the progression of chronic musculoskeletal disorders, including most notably rheumatoid arthritis.

Despite conducting an extensive literature search (of the following data sources from until 31^st^ January 2013: MEDLINE, EMBASE, AMED, CINAHL, CENTRAL, and Google Scholar), we identified only one previous randomised placebo controlled trial on the use of magnet therapy for rheumatoid arthritis. The results of this trial, which compared strong versus weak magnets strapped to the knee, showed that there was no statistical difference in pain outcomes between experimental and control groups [9].

The primary objective of the present trial was to investigate whether the practice of wearing magnetic wrists straps offers any specific therapeutic benefit for patients with rheumatoid arthritis in terms of pain, inflammation, physical function, and overall disease activity. A secondary objective of the trial was to establish whether there are specific therapeutic effects of wearing a copper bracelet for patients with rheumatoid arthritis.

## Materials and Methods

The protocol for this trial is publicly available as a supporting document [10]. The protocol for this trial and supporting CONSORT checklist are available as supporting information; see Checklist S1 and Protocol S1.

### Trial design

The trial used a randomised placebo-controlled double-blind crossover design in which each participant served as his/her own control.

### Participants

Eligible patients were recruited from primary care, had been diagnosed with rheumatoid arthritis, were adults (aged 18 or over), and reported chronic pain (≥3 months duration and ≥30/100 on a pain visual analogue scale) at the time of enrolment.

### Interventions

Participants wore a standard 2212 (SD = 120) gauss bipolar magnetic wrist strap (experimental device), and three control devices. These were: (1) an attenuated (i.e. weak) 303 (SD = 30.3) gauss magnetic wrist strap; (2) a demagnetised (<20 gauss) wrist strap; and (3) a copper bracelet. Each device was worn for five weeks, with each treatment phase being separated by a one week wash-out period.

The desired magnetic strength of the attenuated wrist strap was determined through experimentation. At 250 gauss or above the device would stick to a refrigerator, which was viewed as a prerequisite for adequate blinding. However, it was acknowledged that even a very weak magnet might have some specific therapeutic action, thereby producing a more conservative estimate of treatment effect for the experimental device. We therefore ensured that none of the attenuated devices exceeded 350 gauss to minimise this possibility.

Plain copper bracelets were used with the intention of serving as an additional placebo. In the case of osteoarthritis, at least, research evidence indicates that such devices may be considered as inert [11]–[12].

### Outcomes

Participants completed questionnaires at recruitment and at the end of each treatment period. Pain levels were self-reported using a 100 mm visual analogue scale (VAS) as the primary outcome measure, ranging from “*no pain*” to “*worst pain ever*”, and the McGill Pain Questionnaire [13]. Participants also reported self assessed tender and swollen joint counts [14]. Physical function was assessed using the Disability Index of the Health Assessment Questionnaire [15]. Additional self reported measures included the Arthritis Helplessness Index and the EQ-5D [16]–[17]. Participants attended their doctors' surgery, providing blood samples which were tested for acute phase reactants, C-reactive protein (CRP) and plasma viscosity (PV), thereby providing objective outcome data on inflammation. Disease activity was assessed using a 100 mm visual analogue scale, and a composite measure of objective and self report measures, modelled upon the CRP version of Disease Activity Score [18]. Medication use was monitored using diaries and manual pill counts before and after each treatment period.

### Sample Size

The sample size calculation for this trial indicated that complete primary outcome data from 62 participants would be required to provide 80% power to detect a clinically important difference of 20% in pain outcomes (100 mm visual analogue scale) between devices using a one way analysis of variance (p = 0.05). This assumed a mean outcome score of 65 for the demagnetised device, with a difference of –6.5 for both the attenuated device and copper bracelet, and -13 for the standard magnetic device, together with a common standard deviation within subjects of 21.7. To allow for attrition we aimed to recruit 69 participants.

### Randomisation

Participants were randomly allocated to one of 24 unique treatment sequences, which determined the order in which they wore the four devices. Randomisation was performed remotely by an independent researcher using a computer programme and block sizes of 24 to avoid bias in sequence allocation and minimise the possible influence of any period by treatment interaction on observed outcomes.

### Blinding

Participants were informed that the purpose of the trial was to evaluate the effects of magnetic and copper bracelets, and that one or more of the four devices might be a placebo. Further details about the devices were withheld. Standard, attenuated and demagnetised wrist straps appeared visually identical. Devices were transported in sealed foam padded boxes and participants were instructed to prevent the researcher from seeing devices. Participants, researchers and care providers were all blind to the randomisation sequence.

It was anticipated that some participants would experiment with their devices and discover that one of the wrist straps was not magnetic, which might exaggerate any apparent therapeutic effect of the standard magnetic wrist strap. The demagnetised wrist strap was included partly for this reason, i.e. to help cancel out any specific therapeutic effect associated with the other control devices, and as a dummy device to divert critical attention away from the attenuated wrist strap and copper bracelet as actual placebos, thereby encouraging participants to believe that these latter devices were intended to have some genuine effect.

### Statistical methods

All analyses were conducted using two-sided significance tests, with a P-value of 0.05 selected as indicative of statistical significance. The main method used to compare treatment outcomes for the four devices was analysis of variance (ANOVA); using *proc glm* in SAS®, version 9.1. This involved routine testing for period effects using methods described by Senn (pp.171–8) [19]. Model assumptions were checked prior to analysis, involving log transformation of data where necessary. As a secondary pre-specified step, the analysis of data for the primary outcome measure was adjusted by including medication use as a covariate, and the possibility of treatment by period interactions was also tested.

The four devices were compared against each other using multiple comparison adjustment (Tukey-Kramer method) to produce estimates for the least square mean difference between the standard magnetic wrist strap and each of the three control devices, together with associated confidence intervals.

In the case of missing blood test results, missing values were derived where possible through multiple imputation (using *proc mixed*). This used outcome data reported in post treatment questionnaires, including number of swollen joints and participants' global assessment of disease activity.

All statistical analyses were guided by the principle of intention to treat, with the exception of a secondary pre-specified per protocol analysis.^10^ This took into account the effects of non-compliance in cases where participants failed to wear a particular device for 12 hours or more on average per day (i.e. recommended daily use) by removal of associated outcome data.

### Development of composite measure of disease activity

Pooled or composite measures of disease activity are widely employed and advocated for in trials of rheumatoid arthritis [20]. To provide a more robust outcome measure of treatment effect, we developed a single composite measure of disease activity, modelled upon the latest CRP version of the Disease Activity Score [18], using principle component analysis to examine the correlation coefficients between self assessed baseline measures of tender joints, swollen joints, and disease activity, together with post treatment CRP scores for the demagnetised wrist strap. From this we selected the first principal component coefficient to provide the following formula for the calculation of disease activity scores:
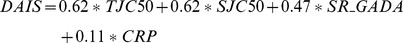
Where:

DAIS = Disease Activity Index Score.

TJC50 = tender joint count [14].

SJC50 = swollen joint count [14].

SR_GADA = self reported global assessment of disease activity (100 mm VAS).

CRP = C-reactive protein.

### Regulatory approval

Full ethical approval for the trial was granted by Hull and East Riding Local Research Ethics Committee in February 2007, together with research governance approval shortly thereafter from Hull Teaching Primary Care Trust (PCT), East Riding of Yorkshire PCT, and York & North Yorkshire PCT. All participants provided informed written consent.

## Results

### Participants

**Figure 1:**
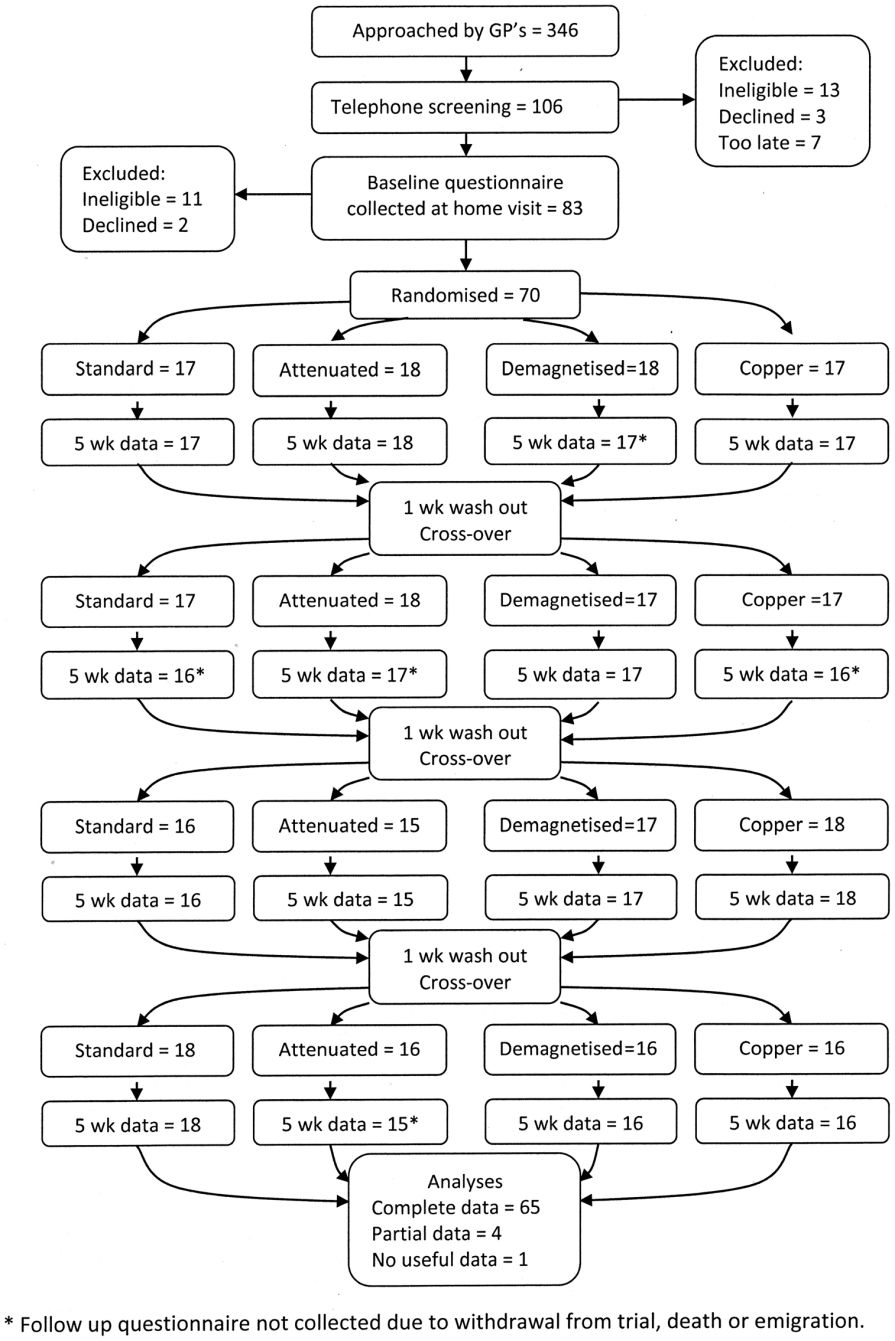
Participant flowchart (CONSORT flowchart). Numbers following randomisation relate to participants and questionnaires.

Patient recruitment began in July 2007 and finished in March 2008. Figure 1 illustrates the flow of patients through the trial, together with the collection of self-reported outcome data. Information about the trial was sent to 346 patients, with verified rheumatoid arthritis, by nine general medical practices across North and East Yorkshire. Of the 106 people who initially volunteered to take part, 24 were excluded because they did not meet eligibility criteria, most commonly because of low levels of pain. Five people declined to take part in the trial after asking further questions, due largely to concerns regarding inconvenience associated with providing blood samples, and seven patients volunteered after recruitment had closed.

In total, seventy patients were recruited and randomly allocated to one of the 24 different treatment sequences, which involving wearing the four devices in turn. Baseline characteristics of all trial participants are shown in Table 1. Participants were aged 33 to 79 years, and were predominantly female. All patients were being prescribed either analgesics, disease modifying anti-rheumatic drugs (DMARDs), or non-steroidal anti-inflammatory drugs (NSAIDs) at the time of recruitment.

**Table 1:** Participant characteristics at recruitment.

*Demographics*		*Outcome measures*	
Age in years	62.0 (12.1)	Pain VAS: 0 to 100	59.0 (21.3)
No. of females	52 (74%)	MPQ – Pain Rating Index: 0 to 78	17.5 (9.7)
Time from first symptoms to recruitment in years	16.3 (11.7)	Tender joint count: 0 to 50	15.0 (11.2)
Time from diagnosis to recruitment in years	14.7 (11.7)	Swollen joint count: 0 to 50	10.3 (11.1)
***Employment status***		Disease activity VAS: 0 to 100	52.7 (23.1)
No. retired	40 (57%)	HAQ – Disability Index: 0 to 3	1.35 (0.67)
No. in paid employment	18 (26%)	Arthritis Helplessness Index: 5 to 30	16.7 (3.4)
***Education***		***Joints affected by pain or swelling*** †	
No. with post-school education	20 (29%)	No. fingers / thumbs	63 (90%)
No. with degree or equivalent qualification	8 (11%)	No. wrists	49 (70%)
***Previous use of CAM***		No. shoulders	42 (60%)
No. who had ever tried a magnetic bracelet	15 (21%)	No. knees	42 (60%)
No. who had ever tried a copper bracelet	29 (41%)	No. toes	40 (57%)
Other therapies: 0 to 15*	1.46 (1.93)	No. ankles	37 (53%)
No. who had ever tried acupuncture	23 (33%)	No. elbows	27 (39%)

Values are means (SD) unless otherwise indicated.

*Mean total (SD) of other therapies tried previously from a list of 15 common forms of complementary or alternative medicine (CAM). Most commonly used other form of CAM listed underneath.

† Determined from self-assessed swollen and tender joint counts at recruitment.

### Anticipated effects of magnetic and copper bracelets

Participants were asked at recruitment to state (on a 5 point scale) if they believed that wearing magnetic or copper bracelets might help to relieve their symptoms (table 2). Analysis of responses showed no significant difference between magnetic and copper bracelets in terms of their expected effects (*df*  = 4, *N* = 70, χ^2^ = 1.66, *p* = 0.80).

**Table 2:** Belief in the power of bracelets to relieve arthritis symptoms.

Magnetic bracelet		Copper bracelet	
Very likely (to relieve symptoms)	1 (1.4%)	Very likely (to relieve symptoms)	1 (1.4%)
Fairly likely (to relieve symptoms)	10 (14.3%)	Fairly likely (to relieve symptoms)	8 (11.4%)
Can't decide	41 (58.6%)	Can't decide	42 (60.0%)
Fairly unlikely (to relieve symptoms)	12 (17.1%)	Fairly unlikely (to relieve symptoms)	9 (12.9%)
Very unlikely (to relieve symptoms)	6 (8.6%)	Very unlikely (to relieve symptoms)	10 (14.3%)

Values are frequencies and (percentage).

### Data collection at follow-up

Sixty five trial participants finished all four phases of the study, and were successfully visited at home after every five week treatment period, thereby providing complete questionnaire data. We were unable to collect complete questionnaire data from five participants. One participant died during the second treatment phase. One other person emigrated from the UK during the final treatment phase and could not be contacted. Three participants withdrew entirely from the trial during the first and second treatment phases for unknown reasons. 266 (out of 280) questionnaires were successfully completed and collected at follow-up. A total of 467 out of 560 PV and CRP blood test results were successfully obtained. Values for a further 65 of the 93 missing blood samples were imputed from available questionnaire data.

Unadjusted mean scores for each outcome measure are shown in Table 3. Table 4 shows the least square mean difference in treatment outcomes between the standard magnetic wrist strap and each of the three control devices, including relevant confidence intervals, together with an overall estimate of statistical significance for differences in outcome across the four devices when adjusted for the effects of period.

**Table 3:** Unadjusted treatment outcomes.

Outcomes (lower is better for all measures)	Devices			
	Standard wrist strap (n = 67)	Attenuated wrist strap (n = 65)	Demagnetised wrist strap (n = 67)	Copper bracelet (n = 67)
***Pain***				
Pain VAS: 0 to 100	48.23 (26.17)	49.98 (26.17)	53.37 (22.23)	53.03 (24.30)
MPQ – Pain Rating Index: 0–78	14.78 (10.54)	15.11 (9.58)	15.87 (10.56)	14.51 (10.49)
Tender joint count: 0 to 50	12.49 (12.24)	14.05 (12.88)	13.52 (13.45)	14.85 (14.12)
***Inflammation***				
Swollen joint count: 0 to 50	8.84 (10.32)	10.20 (13.09)	9.95 (13.52)	9.65 (11.98)
C-reactive protein (n = 60, 60, 60, 57)	11.72 (16.19)	8.95 (17.94)	11.75 (24.24)	12.04 (20.64)
Plasma viscosity (n = 57, 59, 56, 58)	1.72 (0.12)	1.68 (0.10)	1.73 (0.17)	1.72 (0.15)
***Disease activity***				
Disease activity VAS: 0 to 100	48.12 (23.24)	45.57 (24.72)	48.78 (22.90)	50.00 (23.27)
***Physical function***				
HAQ – Disability Index: 0 to 3	1.27 (0.73)	1.26 (0.73)	1.28 (0.72)	1.29 (0.77)
***Helplessness***				
Arthritis Helplessness Index: 5 to 30.	15.15 (3.86)	15.20 (3.75)	15.02 (4.05)	15.08 (4.33)
***Medication use:*** * number of tablets or capsules taken during treatment phase*				
Analgesics	85.50 (90.53)	88.46 (98.74)	76.91 (83.53)	79.26 (91.84)
DMARDS	39.61 (48.25)	40.94 (52.44)	39.09 (47.12)	39.00 (45.69)
NSAIDS	27.59 (41.08)	26.78 (31.44)	27.97 (41.24)	26.12 (33.88)

Values are unadjusted means and (SD).

**Table 4:** Statistical comparison of treatment effects.

Outcomes (negative values indicate superiority of the standard magnetic wrist for all measures)	Devices			*p*
	Attenuated wrist strap (n = 65)	Demagnetised wrist strap (n = 67)	Copper bracelet (n = 67)	
***Primary outcome measure***				
Pain VAS: 0–100	−2.34(−9.41 to 4.73)	−5.14(−12.17 to 1.89)	−5.01(−12.04 to 2.03)	0.18
Pain VAS – added adjustment for medication	−2.31(−9.25 to 4.61)	−5.63(−12.47 to 1.21)	−4.83(−11.74 to 2.07)	0.21
Pain VAS – added adjustment for period * treatment interaction	−2.38(−9.51 to 4.74)	−4.96(−12.04 to 2.13)	−4.98(−12.07 to 2.10)	0.19
Pain VAS – added adjustment for pain or swelling in the wrist	−0.94(−8.60 to 6.73)	−5.89(−13.52 to 1.17)	−4.65(−12.30 to 3.01)	0.14
***Secondary pain outcome measures***				
MPQ Pain Rating Index: 0–78	0.93(0.75 to 1.17)	0.84(0.67 to 1.05)	1.00(0.81 to 1.25)	0.13
MPQ Pain Rating Index – added adjustment for medication	0.94(0.75 to 1.18)	0.82(0.65 to 1.02)	0.99(0.79 to 1.24)	0.08
Tender joint count: 0 to 50	0.81(0.62 to 1.06)	0.92(0.70 to 1.20)	0.84(0.64 to 1.10)	0.17
***Inflammation***				
Swollen joint count: 0 to 50	0.92(0.68 to 1.24)	1.01(0.74 to 1.34)	1.01(0.75 to 1.34)	0.83
C-reactive protein	1.25(0.92 to 1.70)	1.07(0.79 to 1.45)	1.06(0.77 to 1.45)	0.27
Plasma viscosity	0.03(−0.01 to 0.07)	−0.01(−0.05 to 0.03)	−0.01(−0.05 to 0.04)	0.06
***Disease activity***				
Disease activity VAS: 0 to 100	2.13(−4.09 to 8.35)	−0.45(−6.63 to 5.74)	−1.54(−7.72 to 4.64)	0.48
Composite measure	1.04(0.82 to 1.32)	0.95(0.75 to 1.20)	0.92(0.72 to 1.17)	0.52
***Physical function***				
HAQ – Disability Index: 0 to 3	−0.01(−0.12 to 0.10)	−0.03(−0.14 to 0.08)	−0.03(−0.14 to 0.08)	0.86
***Coping***				
Arthritis Helplessness Index: 5 to 30.	−0.03(−0.81 to 0.74)	0.19(−0.58 to 0.96)	0.20(−0.57 to 0.96)	0.80
***General health***				
EQ-5D	0.04 (−0.06 to 0.15)	0.02 (−0.08 to 0.13)	0.05(−0.05 to 0.16)	0.58
***Medication use:*** * number of tablets or capsules taken during treatment phase*				
Analgesics	1.05(0.77 to 1.42)	1.13(0.83 to 1.53)	1.10(0.80 to 1.47)	0.77
DMARDS	0.97(0.85 to 1.10)	0.10(0.88 to 1.13)	1.03(0.91 to 1.16)	0.68
NSAIDS	1.03(0.80 to 1.31)	1.23(0.96 to 1.58)	1.15(0.90 to 1.47)	0.09

Values refer to the least square mean difference in treatment effects between the standard magnetic wrist strap and each of the control devices, using Tukey's HSD test for multiple comparison adjustment (95% CI). *p* is based on ANOVA, adjusted for period.

*Note: HSD = Honestly Significant Difference*.

### Analgesic effects

Analysis of pain outcomes (Pain VAS) by ANOVA, including participant, period, and treatment as factors showed no significant difference amongst the four devices (*p* = 0.18). Adjustment for medication use did not alter this finding (*p* = 0.21). There was no evidence of a period effect (*p* = 0.29) or a period by treatment interaction (*p* = 0.64). There was no statistically significant difference between devices when adjusting for the fact that some participants (30%) reported that their wrists, on which devices were worn, were unaffected by rheumatoid arthritis.

When compared individually, estimated 95% confidence intervals for the true difference in outcomes between devices indicated that, measured on a 100 mm VAS, use of the standard (experimental) magnetic wrist strap may realistically have resulted in anything up to and including a 12 mm reduction in pain, or conversely a 5 mm increase in pain, depending on the comparator device (see table 4). Such differences were not, however, statistically significant.

No statistically significant differences were found overall for the McGill Pain Questionnaire (MPQ) – Pain Rating Index, even when adjusted for medication use (*p* = 0.08). Self assessed tender joint count differences among the four devices were not statistically significant (*p* = 0.17). In addition, estimated 95% confidence intervals for the relative difference between devices for each of these secondary pain measures, failed to show any analgesic benefit resulting from the use of the standard magnetic wrist strap.

### Effects on inflammation

No statistically significant differences among the four devices were observed for self reported and biological measures of inflammation, affected number of joints, CRP and PV test result outcomes (see tables 3 and 4). Replacement of missing data using multiple imputation did not affect these results.

### Disease activity

Analysis of data for self-reported disease activity failed to demonstrate any statistically significant difference between devices (*p* = 0.48). Despite a probable increase in sensitivity and statistical power relating to the development of a composite measure of disease activity, we failed to identify any statistically significant difference amongst the four devices using this measure (*p* = 0.52).

### Physical functioning, helplessness and medication measures

No statistically significant differences were found for the disability index of the Health Assessment Questionnaire or the helplessness subscale of the Arthritis Helplessness Index. Moreover, we were unable to identify any meaningful difference in participants' use of either analgesics, DMARDS or NSAIDs whilst wearing each of the four different devices (see tables 3 and 4).

### Compliance and per protocol analysis

Participants reported that on average they had worn each device for 565 hours (SD = 222 hours), i.e. just over 16 hours per day. Removal of treatment outcome data for cases in which a participant reported wearing a particular device for less than 420 hours in total (12 hours per day), as part of a pre-specified per-protocol analysis, did not affect the results of the trial.

### Safety

#### Serious adverse events

One participant died during the second treatment phase of the trial. The cause of this person's death was attributed to infection of a previously amputated limb with methicillin-resistant staphylococcus aureus (MRSA). Other serious adverse events were also reported, but again these were not related to participation in the study.

#### Minor adverse reactions

Patients were not permitted to participate if they had a known allergy to copper. Despite this, seven participants reported skin irritation caused by the copper bracelet. One other person reported headaches whilst using the copper bracelet and another complained of an unpleasant metallic taste in the mouth.

A small number of adverse reactions were also attributed to the standard and weak magnetic wrists straps. These included minor skin irritation, which might have been caused by nickel in the metal buckle, and dizziness. However, these same reactions were also reported for the demagnetised wrist strap.

### Success of blinding

Success of blinding was assessed by asking participants to indicate on a 5-point Likert scale the extent to which they believed each device was or was not a placebo (table 5). Results obtained failed to show any statistically significant difference in beliefs between the standard magnetic wrist strap and either the attenuated wrists strap (*df* = 4, N = 127, χ^2^ = 2.24, *p* = 0.69) or the copper bracelet (*df* = 4, N = 131, χ^2^ = 2.42, *p = *0.66). However, beliefs did differ significantly in respect of the demagnetised wrist strap, in that more participants believed that this device was inactive (*df* = 4, N = 128, χ^2^ = 11.61, *p = *0.020). Together, this indicated that only the demagnetised wrist strap performed inadequately as a valid placebo in terms of successful blinding.

**Table 5:** Differences in belief as to whether each device was a placebo.

Belief	Devices			
	Standard wrist strap (n = 64)	Attenuated wrist strap (n = 63)	Demagnetised wrist strap (n = 64)	Copper bracelet (n = 67)
Definitely a placebo (i.e. inactive)	3 (4.7%)	4 (6.3%)	11 (17.2%)	3 (4.5%)
Probably a placebo (i.e. inactive)	10 (15.6%)	13 (20.6%)	15 (23.4%)	9 (13.4%)
Can't decide	29 (45.3%)	27 (42.9%)	29 (45.3%)	25 (37.3%)
Probably not a placebo (i.e. active)	11 (17.2%)	13 (20.6%)	6 (9.4%)	19 (28.4%)
Definitely not a placebo (i.e. active)	11 (17.2%)	6 (9.5%)	3 (4.7%)	11 (16.4%)

Values are frequency (percentage).

## Discussion

### Principal findings

The results of this trial indicate that participants with rheumatoid arthritis obtained little if any specific therapeutic benefit from magnet therapy, involving the use of a 2200 gauss magnetic wrist strap for just over one month. The experimental wrist strap, which was typical of other commonly available devices as regards its magnetic properties and method of application, did not appear to outperform: (a) a very weak (300 gauss) magnetic wrist strap; (b) a non-magnetic wrist strap; or (c) a copper bracelet. Whilst estimated 95% confidence intervals for the individual comparison of experimental and control devices indicate that use of the standard magnetic wrist strap may have resulted in a modest reduction in pain, equivalent to 12 mm on a 100 mm pain VAS, they also indicate the possibility that use of this device may have resulted in a slight increase in pain. Despite such uncertainty, these differences may be viewed as small in terms of potential clinical relevance, and further results obtained for secondary pain measures failed to indicate any analgesic benefit whatsoever resulting from magnet therapy. No overall statistically significant differences were found between experimental and control devices for the primary pain outcome measure (i.e. pain VAS), the McGill Pain Questionnaire, self-assessed measures of tender and swollen joints, disease activity status, physical function, feelings of helplessness, or for two different blood tests used for monitoring levels of acute phase reactants as indicators of bodily inflammation, even when controlling for medication use, local rather than systemic inflammation, and non-compliance. Similarly, we did not observe any evidence, of statistical significance or likely clinical importance, to suggest superiority of the copper bracelet over other control devices.

### Strengths and limitations

The main strength of this trial was the use of multiple comparators, consisting of two placebos and one dummy device, which controlled for non-specific effects that may arise in studies of magnet therapy due to inadequate blinding and differences in treatment expectancy. Had the trial relied exclusively on a single demagnetised comparator then this might have cast doubt on findings, due to observed difficulties of blinding. In addition, the use of a randomised crossover design ensured that the sample size was sufficiently large to provide the statistical power to detect a relatively small difference in treatment effects. The recruitment target was exceeded and attrition was lower than anticipated. The high questionnaire completion rate was almost certainly attributable to the use of home visits at follow up. A wide range of possible therapeutic outcomes were assessed, including ‘hard’ outcome data on inflammation, provided by the collection and testing of blood samples. These were established biological measures used routinely for monitoring changes in disease activity for patients with rheumatoid arthritis, which may be less affected by non-specific factors than self-reported outcomes [21]. We also incorporated wash out periods as a safeguard against the possible influence of carry over effects, the absence of which appears to be supported by results obtained.

In terms of potential limitations, one disadvantage of crossover designs is that they are not well suited to lengthy periods of treatment or follow-up. It is therefore possible that devices were not worn for long enough in order to produce a meaningful treatment effect. However, the fact that the standard magnetic device used in this trial was sold commercially with a 30 day satisfaction or money back guarantee suggests that a 5 week treatment programme should have been adequate. Instructions accompanying the device also recommended a minimum of eight hours daily use. Yet, participants reported that on average they had worn devices for more than twice this duration each day. Moreover, conduct of a secondary per protocol failed to alter the findings.

Another theory that could explain the apparent absence of any meaningful difference in treatment outcomes between devices would be that magnet therapy has local rather than systemic treatment effects. Inclusion of patients whose wrist joints were not badly affected by rheumatoid arthritis might therefore be questioned. However, the vast majority (i.e. 70%) of trial participants did report pain or swelling of the wrists and adjustment for this variable did not affect the results.

### Comparison with previous research findings

The present trial is unique in being the first randomised control trial to investigate the effects of magnets worn on the wrist for patients with rheumatoid arthritis. Indeed, only one previous trial has been reported concerning the use of magnet therapy for rheumatoid arthritis, in which 64 participants wore either 1900 gauss or 720 gauss magnets on the knee for a period of one week [9]. Although the present trial had greater statistical power, involved a much longer period of treatment, and used more appropriate placebo control methods, results obtained from both trials appear generally consistent in showing that a standard magnetic device had no demonstrable effect on pain, inflammation or physical functioning, beyond that of a placebo.

### Meaning of the study

The results of this study may be understood in a number of ways. The most obvious interpretation is that they demonstrate that magnetic wrist straps, and also copper bracelets, have little if any specific therapeutic effects (i.e. beyond those of a placebo) on pain, inflammation, or disease activity in rheumatoid arthritis.

Both the outcome measures and sample size selected for this trial were determined from guidelines issued by the *American Colleague of Rheumatology* (ACR) [22]. These specified a minimum benchmark of 20% improvement in five out of seven ‘core’ measures as indicative of a clinically meaningful response to treatment. The fact that we were unable to demonstrate such a difference for the primary outcome measure on its own, nor indeed any of the other core measures employed, strongly suggests that wearing magnetic wrists straps, or copper bracelets, in order to minimise disease progression and alleviate symptoms of rheumatoid arthritis is a practice which lacks clinical efficacy.

An alternative, although somewhat less convincing interpretation, is that the trial was underpowered and the results are inconclusive. In particular, critics may point towards a seemingly small sample size and the risk of committing a type II error through over reliance on data obtained for a pain visual analogue scale, as a relatively crude and insensitive primary outcome measure.

Certainly, a sample of just 70 participants may appear too small for an adequately powered randomised controlled trial. Yet, in comparison to parallel group designs, crossover trials provide much greater statistical power, because each participant acts as his/her own control, thereby reducing error variance. However, whilst the number of people recruited and successfully followed up in this study was in fact exceptionally large for a crossover trial [23], the authors do acknowledge that the present study cannot be considered as definitive.

Estimated confidence intervals for the difference between devices measured using a 100 mm pain VAS may be viewed as quite broad and include a possible reduction in pain of up to 12 points in favour of the standard magnetic wrist strap. This almost reaches a level specified as clinically important within original sample size calculation.

Differences for the pain VAS were not however found to be statistically significant. Moreover, it was precisely because of our own concerns regarding the measurement properties of a simple VAS, and to a lesser extent the self-assessed measure of tender joints, that we included a third measure of pain, i.e. the McGill Pain Questionnaire, which did not feature in the ACR recommendations. It is therefore worthwhile noting that, results for these secondary pain measures do not indicate any therapeutic superiority, statistically significant or otherwise, of the standard magnetic wrist strap over the other devices included in this study.

Importantly, since this study was first conceived, the American College of Rheumatology and the The European League Against Rheumatism have issued joint guidance on and reporting of trials concerning rheumatoid arthritis [24]. This favours the use of a simplified index-based measure of disease activity, such as that of the composite measure reported in the present paper. This has the advantage of providing a reliable and valid indicator of remission and its use is therefore preferable to exclusive reliance upon any single ‘primary’ outcome measure. However, results obtained for this composite measure, as shown in Table 4, again show no statistically significant difference between the four devices employed in this trial.

Although it would be inappropriate to infer equivalence between devices based purely on the apparent absence of any statistically significant difference, as measured using a simple pain VAS, the overall consensus of results obtained for the various outcome measures employed within this trial, together with their varying degrees of precision, does tend to suggest that the therapeutic effects of the standard magnetic wrist strap, and also the copper bracelet, may be considered as broadly similar, if not the same, as those of a placebo.

Whilst replication is advisable, findings from the present trial offer little encouragement for future research concerning the therapeutic efficacy of either magnetic or copper bracelets for rheumatoid arthritis. Given inherent problems of blinding, future trials should seek to employ equally rigorous control methods to counteract the untoward influence of non-specific factors. In particular, the results of this study demonstrate that research participants can readily distinguish between magnetic and non-magnetic devices. Therefore, sole reliance on demagnetised facsimiles as comparators should be avoided in future placebo controlled trials of magnet therapy.

The findings of this study do not refute the view that both magnetic and copper bracelets are inexpensive, generally safe, or indeed that wearers may perceive some analgesic benefit, albeit if due to psychological placebo effects or regression fallacy. However, the promotion and use of such devices for arthritis may present a significant opportunity cost. In the case of rheumatoid arthritis, early diagnosis and suppression of inflammation is essential to minimise joint damage and disability [25]. Yet, the results of this trial fail to provide any support for belief in the anti-inflammatory effects of magnetic wrists straps and copper bracelets. Similarly, the devices used in this trial did not appear to alter disease activity. Therefore, people with rheumatoid arthritis who chose to wear such devices rather than seeking early diagnosis and effective treatment may be risking their health.

## Conclusions

The overall findings of this trial indicate that magnetic wrist straps, and also copper bracelets, have no statistically significant, nor clinically meaningful, therapeutic effects upon rheumatoid arthritis. The devices worn in this trial offered little if no specific benefits, i.e. beyond those of a placebo, in reducing pain, inflammation, disability, disease activity, and medication use amongst this patient group.

## Supporting Information

Checklist S1 **CONSORT statement.**
(DOC)

Protocol S1 **Trial Protocol.**
(DOC)
