# Supplementary material for: Copper Bracelets and Magnetic Wrist Straps for Rheumatoid Arthritis – Analgesic and Anti-Inflammatory Effects: A Randomised Double-Blind Placebo Controlled Crossover Trial
Source: PLoS One. 2013 Sep 16;8(9):e71529. doi: 10.1371/journal.pone.0071529 (PMC3774818; doi:10.1371/journal.pone.0071529)
Supplement: Protocol S1 — Trial Protocol. (DOC) [file pone.0071529.s002.doc]

**CAMBRA Trial Protocol**

**Stewart Richmond**

October 2006

1. **TRIAL IDENTIFIER**
   1. **Full title of trial**

Magnet therapy for the relief of pain and inflammation in rheumatoid arthritis: a randomised double-blind placebo-controlled trial.

- 1. **Acronym**

CAMBRA (Copper And Magnetic Bracelets for Rheumatoid Arthritis)

- 1. **Chief investigator**

Stewart Richmond - Research Fellow

1. **THE NEED FOR A TRIAL**
   1. **What is the problem to be addressed?**

The proposed trial will investigate the therapeutic effectiveness of magnet therapy when used as an adjunct to practitioner led management of pain and inflammation in rheumatoid arthritis (RA). Possible effects on other major health outcomes will also be considered. This will involve a comparison of treatment effects between a commercially available magnetic device and three control devices, which are intended to act as placebos. This addresses a need for rigorous scientific evidence on the subject.

- 1. **What is the principal research question to be addressed?**

The primary objective of this trial is to determine whether or not a widely available permanent static magnetic device (PSMD) provides any true therapeutic benefit for patients with RA in terms of pain and inflammation.

- 1. **What are the secondary research questions to be addressed?**

Secondary objectives of this trial are:

- to determine the actual therapeutic effectiveness of a PSMD in terms of the following additional outcomes:
  - functional status and disease activity
  - general health
  - quality of life
  - use of analgesic medication
- to identify specific factors that may predict actual therapeutic benefit derived from magnet therapy in terms of major outcomes.
- to identify any specific factors which mediate observed placebo effects.
- to report any adverse events or reactions relating to the safe use of devices employed.
- to gather economic data which may be used to evaluate the cost-effectiveness of magnet therapy.
  1. **Why is the trial needed now?**

Rheumatoid arthritis (RA) is a chronic inflammatory autoimmune disease which typically begins to develop between 30 to 50 years of age. It is estimated that 387,000 people in the UK have RA, with three times more women affected than men. RA involves periodic inflammation of the synovium in joints of the hand, wrist, foot, knee or shoulder. This causes swelling of the joint capsule and irritation of nerve endings, producing pain and resulting in damage to both bone and cartilage. In turn this may lead to both disability and mortality (ARC 2002, ARC 2003). Favoured diagnostic criteria are those of the American College of Rheumatology (Arnett et al 1998).

The burden which RA imposes upon both the NHS and the economy in general is considerable. It is reported that over just a one year period (1999 to 2000) inflammatory arthritis accounted for 1.9 million GP consultations, 45,887 hospital admissions and a loss of £833 million in productivity (ARC 2002). For the individual the consequences of RA may be devastating.

Treatment of RA is currently dominated by the use of medication. Non steroidal anti-inflammatory drugs (NSAIDs) are the most widely used pharmacological agents, although 2^nd^ generation anti-inflammatories in the form of Cox-2 inhibitors may be preferable (Madhok et al 2000). Patients with RA are also frequently prescribed disease modifying anti-rheumatic drugs (DMARDs). Whilst recently developed biological agents in the form of TNF-α antagonists have been shown to prevent damage (Emery 2006). Whilst such drugs may alter disease progression, people with arthritis rarely find that they are entirely adequate for the purpose of pain control. Therefore analgesics are also typically prescribed as an adjunct (Jordan et al 2004).

The fact that all commonly prescribed drugs for rheumatoid arthritis are associated with unwanted side effects is worrying for both practitioners and patients. For example, DMARDs are associated with high levels of toxicity and therefore cannot be tolerated for long periods (Emery 2006). Moreover, NSAIDs have been shown to cause gastrointestinal ulcers, which may result in perforation, bleeding and death (Rodriguez and Jick 1994, Madhok et al 2000, Walker-Bone et al 2000).

Over recent years health professionals have witnessed a patient led revolution in the use of complementary and alternative medicine (CAM). Whilst reliable estimates for CAM use are hard to obtain, results of a recent population based survey indicated that around 28% of the UK adult population used some form of CAM in just one year (Thomas et al 2001). However, it has also been shown that people who experience chronic pain are twice as likely to try CAM (Astin 1998). One might therefore anticipate that patients with RA would be included amongst this group. Indeed, this appears consistent with suggestions that almost 60% of people with arthritis use complementary therapy (ARC 2000). In part, such popularity may also be attributed to the perception, albeit sometimes misguided, that complementary therapies lack side effects and therefore represent a safe and ‘natural’ alternative to drugs (Madhok et al 2000, Ernst 2004). Alternatively this may simply relate to a rise in holistic attitudes (Astin 1998, House of Lords 2000).

Whatever the case, such treatments have typically been viewed with scepticism by the medical community. Yet assumptions which directly equate lack of previous scientific evidence to lack of therapeutic efficacy should be challenged. Rather attention should be drawn to the fact that until recently CAM accounted for less than 1% of total research budgets (Wider and Ernst 2003). A figure which is grossly disproportionate to the measurable contribution which CAM makes towards first-contact primary care (Thomas et al 2001). With a growing emphasis on patient choice (NHSE 1998) CAM is now being recognised as a largely untapped resource which offers the potential for therapeutic innovation within the NHS. This view is supported by both the House of Lords and the UK government, which have recommended a major increase in funding for CAM research (House of Lords 2000; Department of Health 2001).

Perhaps one of the most intriguing yet under researched forms of CAM is that of magnet therapy. Quite simply it involves the application of magnetic materials on or very close to the skin over prolonged periods of time. This encompasses a wide range of interventions involving different types of devices, different strength magnetic fields and different modes of administration (Hinman 2002). For example, devices may be unipolar, in which case only one magnetic pole faces towards the skin, or bipolar when two or more magnets are used which face in opposite directions (Trock 2000). However magnet therapy should not be confused with pulsed magnetic field therapy, which involves brief exposure to much stronger electromagnetic fields.

Fascination with the potential health benefits of magnetic forces dates back to antiquity, although in recent times this topic has received a resurgence of public interest (Basford, 2001). Magnet therapy now appears to be one of the most widely used forms of CAM for the management of chronic pain associated with musculoskeletal disorders such as RA, with many patients demonstrating willingness to privately purchase permanent static magnetic devices (PSMDs). Indeed, it is estimated that the worldwide market in PSMDs accounts for somewhere between $2 and $5 US billion (Weintraub 1999, Aspy et al 2002).

Whilst such devices are widely claimed to help alleviate pain for a range of disorders, there appears to be no clearly identified mechanism of action, although theoretically this might involve a reduction in inflammation (Trock 2000). Scientific evidence relating to effectiveness of magnet therapy is however reported to be equivocal (Finegold and Flamm 2006). This has lead to calls for larger and more rigorous randomised controlled trials to determine the actual therapeutic effectiveness, safety and cost-effectiveness of magnet therapy for specific pathological conditions (Ratterman et al 2002; Hinman, 2002).

To the authors’ knowledge only one RCT has yet to focus specifically on the effects of magnet therapy for patients with RA. Segal and colleagues (2001) randomly allocated 64 patients with RA of knee to wear either a bipolar magnetic device (MagnaBloc^TM^) or a seemingly identical unipolar device. The experimental device consisted of four magnets disks (each 190mT) whereas the control device included just one magnetic disk (72mT) and three non-magnetic disks. Devices were attached to the knee for a period of one week in both the treatment (n= 38) and control (n = 26) groups. Both participants and researchers were reportedly blind to treatment allocation. Although participants allocated to the MagnaBloc^TM^ group demonstrated a statistically greater reduction in pain throughout the intervention phase than those in the control group, the study failed to demonstrate a statistically significant difference in pain between the two groups at the end of the 1 week period (p = 0.23). In terms of surrogate measures of inflammation (C-reactive protein and erythrocyte sedimentation rate) no difference was observed between treatment and control groups. In the absence of any evaluation of success in blinding, it is interesting to note participants global assessment of disease activity improved to a significantly greater extent in the treatment group than in the control group (p < 0.01).

Whilst results presented by Segal and colleagues may be challenged on the basis of poor trial design and lack of statistical power, this study nevertheless highlights a number of other methodological weaknesses which characterise other published trials of magnet therapy. Importantly, medication use was not measured although participants were likely to alter their use of analgesics as a response to changes in pain intensity. Any true benefit derived from magnet therapy may therefore have been masked by a reduction in medication use. Trials of magnet therapy should also give participants the opportunity to use devices in a manner which reflects common practice within the real world. Since magnet therapy is generally understood to involve prolonged periods of exposure to magnetic fields, it may be argued that short periods of exposure will prove insufficient for any cumulative therapeutic effect to occur. In the case of the trial described above, an exposure period of one week may well have been inadequate. Certainly compliance should also have been reported since there is no guarantee that trial participants actually wore the devices as recommended. Even if analysis of data is by intention to treat rather than by active treatment, this information will still be of value.

Perhaps the greatest methodological challenge faced by trial of magnet therapy however is that of providing adequate control devices against which any ‘true’ effects from magnet therapy may be assessed. RCTs which incorporate a placebo control group are generally perceived as methodologically superior to those which do not. This is especially the case for trials involving new treatments and subjective outcome measures (Avenell et al 2004, Fergusson et al 2004). The key methodological advantage of placebo controlled trials over open trial designs relates to the ability to blind participants, clinicians and researchers to the allocation of experimental and control treatments (Vickers and de Craen 2000). This helps to avoid systematic bias resulting from differences in the perceived or desired effectiveness of treatments which may alter the outcomes being assessed (Schulz and Grimes 2002, Campbell 2003, Akobeng 2005).

For trials of magnet therapy successful blinding is hampered by the fact that magnetism is a necessary quality of experimental devices which can in most cases be detected with relative ease. Trials which report superior patient outcomes for a magnetic device as opposed to a non-magnetic control device are therefore likely to be criticised on the basis that results may be attributable to non-specific (placebo) effects (Pope and McNally 2002, Patterson and Dieppe 2005). This highlights the need to evaluate success of blinding (Fergusson et al 2004).

The problem of blinding is not necessarily without solution however. One approach is to use an attenuated control device, which retains a weak magnetic force and therefore helps to prevent participants and researchers from correctly identifying the device as a placebo or sham (e.g. Segal et al 2001, Harlow et al 2004). The obvious limitation of this approach however is that rather than being completely inert the control device may have some therapeutic action. As such, this may produce a more conservative estimate of treatment effect between groups. An alternative approach, yet to be reported, is to use a non-magnetic comparator which is marketed commercially as having similar therapeutic benefits, but which in actual fact is ineffective. Such a device would need to have face validity and be used in a similar fashion to the experimental magnetic device. In this sense copper bracelets may be ideal.

Whilst research findings point towards serum copper imbalances in patients with arthritis (Grennan et al 1980, Youssef et al 1983, Milanino et al 1993) it is likely that this reflects a natural metabolic response, rather than a cause, of inflammation (Weber 1984). The link between dermal copper absorption and pain relief in arthritis may therefore be rather tenuous. However, given the popular use of copper bracelets it is therefore surprising that no rigorous scientific research appears to have been conducted on the therapeutic effectiveness of such devices.

In the most widely cited study on this topic Walker and Keats (1976) randomised 240 arthritis sufferers into three groups (see also Walker et al 1981). Group 1 wore a copper bracelet for one month and then an aluminium bracelet for a further month. Group 2 wore identical devices but in reverse order and Group 3 wore no device. From this Walker and Keats reported that significantly more participants rated the copper bracelet as superior than the aluminium bracelet and that copper bracelets actually lost weight by an average of 13mg/month. This appears to support the theory that copper may be leached into the skin and that this may have had a positive therapeutic effect on arthritis symptoms. However, Walker and Keats failed to demonstrate any positive correlation between the weight loss of individual bracelets and perceived efficacy. Major criticisms of this study centre on selection bias and measurement error. For example, 163 or 68% of all participants were excluded from the analysis. Some of whom were removed due to poor compliance, whilst others were excluded on the grounds that their bracelets actually gained weight. Certainly the finding that bracelets included in the analysis lost weight is not therefore surprising. Furthermore, blinding is likely to have been compromised since the control bracelet would have appeared different in weight, colour and corrosion. Indeed, such findings have yet to be replicated elsewhere.

Seemingly contradictory evidence rejecting a link between dermal copper absorption and pain relief in arthritis comes from Shackel and colleagues (1997). This involved a randomised double-blind trial of copper-salicylate versus placebo gel for pain relief amongst 116 participants with osteoarthritis. Whilst one would expect copper to be absorbed more readily in a gel preparation than would be the case with a copper bracelet, findings from this trial failed to demonstrate any difference between the copper-salicylate gel and the placebo gel in terms of pain relief, ratings of treatment efficacy or use of medication after 1 month of treatment. Whether these findings could be generalised to patients with RA is however open to question.

Overall, it has been shown that RA is a common inflammatory musculoskeletal disease which represents a significant burden to the UK health system and the economy. For the individual its effects are both distressing and disabling. Conventional drug treatments for RA may fail to control pain and present a risk to the individual. Increasingly therefore patients with arthritis may be turning to complementary and alternative therapeutic approaches to pain management, such as magnet therapy. However historical underinvestment in CAM research has resulted in a lack of scientific evidence concerning the therapeutic effectiveness of such devices. Further pragmatic research within this field may therefore lead to innovative therapeutic advances in the cost-effective management of diseases such as RA. Within this context the proposed trial will seek to offer a meaningful scientific evaluation of the true therapeutic value of magnet therapy for RA using a novel methodological approach to overcome some of the inherent challenges encountered by research in this field.

- 1. **Systematic reviews**

A review of scientific databases (Cochrane Library, Medline, AMED and CINAHL) confirms an overall paucity of rigorous scientific research into the therapeutic effectiveness of magnet therapy for RA, or indeed any other condition. As such, this search failed to reveal any systematic reviews on the topic, although a number of ‘critical’ reviews were identified.

Hinman (2002) reviewed findings from 14 RCTs and repeated measures design studies involving magnet therapy and pain as an outcome in humans. Of these, 6 demonstrated a statistically significant reduction in pain following magnet therapy, with a further 3 studies showing a non-significant reduction in favour of magnet therapy. More recently, Eccles (2005) reported a review of 21 RCTs, 13 of which demonstrated statistically significant analgesic effects associated with magnet therapy. This review also showed a tendency for higher quality trials to show positive effects. Inconsistencies in findings between trials may be attributed to a variety of factors including variability in methodological quality, differences between device investigated and clinical heterogeneity between conditions studied.

As part of an allied research programme a systematic review of magnet therapy for chronic pain is planned. This will be of direct relevance to the proposed trial.

- 1. **Other relevant trials currently being undertaken**

A search of The Cochrane Central Register of Controlled Trials (CENTRAL) and the *meta*Register of Controlled Trials (*m*RCT) revealed no RCTs of magnet therapy for arthritis currently in progress, apart from that of the MACROPOD trial.

MACROPOD (ISRCTN 18518978) is a randomised double-blind placebo controlled crossover trial of magnet therapy for pain relief in osteoarthritis, which is currently at the analysis stage. In total 45 participants were randomly allocated to one of four treatment sequences consisting of four phases, with each phase lasting one month. Participants each wore a MagnaMax^TM^ bipolar bracelet (mean strength 200.9mT, SD 17.7), an attenuated MagnaMax^TM^ (45.3mT, SD 12.0), a non-magnetic MagnaMax^TM^ and a copper bracelet. Outcomes measures included a 10cm pain visual analogue scale, WOMAC, McGill Pain Questionnaire, SF-36, EQ-5D together with economic items. Medication use was measured by triangulation of diary records, prescription records and by manually counting remaining tablets and capsules before and after each phase. Whilst originally developed as a pilot study for future trials of magnet therapy, MACROPOD is nevertheless fully powered for the purpose of demonstrating a meaningful clinical difference between the commercially available PSMD employed and the three ‘placebo’ devices in terms of the primary outcome. Findings from this study are due to be published towards the end of 2006.

- 1. **How will the results of the trial be used?**

Further research on this topic would seem wise given that conclusive findings either way was would prove beneficial. If effectiveness can be demonstrated then magnet therapy may become a valuable addition to clinical practice, improving quality of life amongst patients and lowering demand for both consultations and specialist referrals. Moreover, it may serve to reduce reliance on potentially harmful drug treatments, thereby preventing unnecessary adverse events. In economic terms it is also worthwhile noting that one-off costs of providing magnetic devices are likely to be far lower than those of repeatedly prescribing analgesic medication. Therefore if, for example, drug prescription costs for arthritis and related conditions could be reduced by just 1% then this would result in an annual saving to the NHS of roughly £3.4 million per annum (ARC 2002). Conversely, if this trial demonstrates that magnet therapy is ineffective then this may serve to educate patients and prevent unnecessary private expenditure. Perhaps more importantly however the present study addresses an immediate need for guidance which will benefit health care professionals and assist in making appropriate health policy decisions.

1. **THE PROPOSED TRIAL**
   1. **What is the proposed trial design?**

The trial will employ a randomised crossover design. Therefore each participant will act as his or her own control. The principle advantage of a crossover design is that error variance is reduced, thereby minimising the sample size required. Crossover designs are also extremely well suited to exploratory research of new treatments (Senn 2002).

- 1. **What are the planned trial interventions?**

Given the inherent difficulties in developing a ‘perfect’ placebo for magnet therapy, this study will build upon methodology employed within the MACROPOD trial by incorporating three distinct control devices. This represents a comprehensive and concerted attempt to overcome different limitations associated with each individual device in terms of blinding and potential therapeutic action. All participants will therefore undergo four treatment phases each lasting for a period of five weeks:

**Phase A: Full strength MagnaMax^TM^ (experimental device)**

The device worn during this phase will be a commercially available bipolar magnetic wrist strap which is marketed for the purpose of relieving pain in conditions such as rheumatoid arthritis. A number of manufacturers have been approached, one of which has agreed to supply their device (MagnaMax^TM)^ at a discounted rate. Magnetic strength will be checked using a calibrated Hall effect probe prior to distribution. However this device was also used in the MACROPOD trial, and is known to have maximum surface field strength of between 180-220mTesla which is common for devices of this type.

**Phase B: Attenuated MagnaMax^TM^ (placebo)**

This device will be identical to the experimental device with the exception that the magnetic field strength will be reduced to between 25 to 35 mT. This will be confirmed using a Hall effect probe. Participants are unlikely to identify this as a placebo, although it is possible that the weak magnetic field produced may have some therapeutic effect.

**Phase C: Demagnetised MagnaMax^TM^ (dummy)**

This device will appear identical to the experimental device, although it will have no magnetic properties. This device should therefore be entirely inert. Participants are likely to realised that device has been demagnetised and therefore view it as a placebo.

**Phase D: Copper bracelet (placebo)**

Participants will also test a commercially available copper bracelet. As copper bracelets are widely used for pain relief by the general public it is likely that participants may expect this to have some therapeutic benefit. Whether or not this device actually represents a valid placebo is a question which will be addressed as part of the trial.

Participants will be asked to wear each of these devices for a minimum of 12 hours per day, although ideally they should be worn continuously.

- 1. **What are the proposed practical arrangements for allocating participants to the trial groups?**

Participants will randomly allocated to 1 of 24 possible treatment sequences. Treatment sequences will consist of four phases, with each phase corresponding to a particular device (as shown in Appendix 1). This method is preferred over the use of a Latin square design, as this will help to minimise possible analytical bias associated with attrition.

Randomisation will be performed remotely and independently by the York Trials Unit using a relevant computer programme to avoid any bias in sequence allocation. Block randomisation will be unnecessary. The randomisation code will be will be concealed from the chief investigator until all data collection has been completed. Once the full randomisation sequence for all potential participants has been determined, then devices will be packed and sealed in identical padded boxes, distinguishable only by a trial ID number and order in which they are to be distributed.

- 1. **What are the proposed methods for protecting against other sources of bias?**

Several additional methods will be employed to counter potential threats from bias in terms of both the internal and external validity.

**Blinding of participants**

The trial will be presented as study to evaluate the effectiveness of magnetic and copper bracelets on arthritis symptoms. All communication will simply state that one or more of the devices being tested might be intended to act as a placebo.

**Blinding of researcher**

Devices will be independently packaged and sealed within plain white padded boxes by the York Trials Unit. Boxes will simply bear the trial ID number and order of distribution. Devices will then be distributed by the researcher at recruitment and at each follow-up. Each box will contain unique laser labels bearing the trial ID number and the mark of an official stamp. Devices will then be worn for a period of 5 weeks. Participants will be instructed to remove the device immediately before each planned follow-up and to seal the device within the padded box, using the labels provided. Devices will be collected by the researcher at the very end of each visit (i.e. after data collection) and returned to the trials unit. The integrity of each seal will be independently inspected and recorded. Participants will also be instructed in writing to avoid describing the device(s) they have tested in any communication with the researcher. This will help to ensure that researchers remain blind to treatment allocation and that all devices are returned, preventing possible contamination of treatment effects.

**Blinding of health care staff**

GPs, practice nurses and phlebotomists will be provided with similar information about the trial to that given to patients. At no point will they be informed which of the four devices are intended to act as placebos. This will help to maintain blinding amongst participants. Members of staff from participating practices who take part in focus groups before the trial begins will be provided with additional information about the devices involved. These individuals will be asked to refrain from discussing this information with colleagues or patients.

**Stratified random sampling**

Of those practices which express willingness to participate in the trial, nine practices will be selected initially. Ideally three practices will be selected within each trust. This process will be repeated again if necessary in order to include a further three practices. Should the number of potentially eligible patients identified within each practice exceed the target number to be approached (i.e. 24 per practice) by more than 50% (i.e. 36) then patients will be selected at random to receive information about the trial. In such case, patient recruitment will involve stratified sampling according to GP practice. Overall this should help to achieve a fairly representative cross section of patients with RA which will add to the generalisability of findings.

**Wash out-periods**

Whilst randomisation of participants to different allocation sequences should prevent bias associated with potential carry over effects, wash-out periods between treatments will nevertheless be employed. As such, each treatment phase will be separated by a period of one week during which no device will be worn. This should provide additional confidence relating to the internal validity of findings and will aid in the analysis of data (Senn 2002).

- 1. **What are the planned inclusion / exclusion criteria?**

**Inclusion criteria**

- 18 years of age or over
- Diagnosis of rheumatoid arthritis within medical records
- Currently prescribed analgesic medication
- Chronic pain: either persistent or intermittent over a minimum period of three months prior to recruitment (Argoff 2002)
- Current pain: greater than 30/100mm on VAS within the last 24 hours despite medication.

**Exclusion criteria**

- Pregnancy
- Pacemaker or similar device
- Not responsible for administering his/her own medication.
- Dementia or memory impairment, either documented in medical records or if suspected indicated by a score of 6 or below on the Abbreviated Mental Test (Wong et al 2004).
- Diagnosis of malignant disease within medical records
- Forthcoming orthopaedic surgery or analgesic injections within the next 25 weeks.
- Known allergy to copper.
- Regular use of a magnetic or copper bracelet at recruitment
  1. **What is the proposed duration of treatment period?**

Participants begin the first treatment phase immediately after recruitment. Participants will undergo four treatment phases, each lasting five weeks. Each treatment phase will be separated by a one week ‘wash out’ period. Total duration for participation therefore will be 23 weeks.

- 1. **What is the proposed frequency and duration of follow up?**

Baseline questionnaire data will be collected at recruitment. Participants will then be followed up on four further occasions, separated by intervals of six weeks. These will occur at the end of each treatment phase. Blood samples should be taken within a two day period either side of each planned follow-up.

- 1. **What are the proposed outcome measures?**

The recommended approach for assessment of outcomes in clinical trials of treatments for RA is to examine change according to a ‘core set’ of disease activity measures defined by the American College of Rheumatology (ACR). This consists of seven measures. Three of these are self report measures; pain, physical function and global assessment of disease activity. Three rely on clinical examination; swollen joint count, tender joint count and professional assessment of disease activity. With the final measure involving serum analysis of acute phase reactants. (Arnett et al 1988, Felson et al 1993, Felson et al 1995, Rindfleisch and Muller 2005).

For the proposed trial three of these measures would require repeated physical assessment by a suitably qualified clinician, generally speaking a rheumatologist. However this is neither feasible nor warranted. Indeed, Pincus (2005) has shown that a pooled index of the three self-report measures may discriminate equally well to the ‘core set’ in terms evaluating the efficacy of treatments in placebo controlled trials. Perhaps more importantly however the primary focus of this trial is on the effects of magnet therapy on pain and inflammation in RA rather than overall disease activity. As such, the primary outcome measure should be pain specific.

For the present trial a 100mm pain visual analogue scale (VAS) was selected, as consistent with the recommended method of pain measurement according to ACR criteria. This displays verbal anchors indicating “no pain” at one end to “worst pain ever” at the other. Visual analogue scales have been widely used for quantifying pain for a range of conditions including rheumatoid arthritis and have been shown to be adequate for this purpose in terms of reliability, validity and sensitivity (Malaise and Franchimont 1987, Duncan 1989, Bellamy et al 1999). The McGill Pain Questionnaire will be used as a second pain outcome measure, as this covers affective components and may also be used to validate findings from the VAS. This will also aid comparison of findings with studies reported elsewhere.

Possible variation in participants’ use of medication may have a confounding effect on pain outcomes. This will have the tendency of reducing any otherwise observable difference between groups resulting from a true treatment effect. Medication use during each treatment phase will therefore be quantified. This will enable pain outcomes to be adjusted if necessary on the basis of regression analysis. Whilst the main emphasis will be placed on measuring changes in the use of prescribed analgesic drugs (e.g. co-codamol and paracetamol), it is important to note that use of other drug types (e.g. NSAIDs) will have an impact on pain and therefore will be accounted for also.

Mechanisms involved in the alleviation of pain in RA include reduction in inflammation of the synovium. Widely used surrogate measures of inflammation include blood tests, typically C-reactive protein (CRP) and erythrocyte sedimentation rate (ESR). These are referred to by the ACR as acute phase reactants. Together these represent favoured outcome measures for monitoring disease activity (Felson et al 1995). Results presented by Segal and colleagues (2001) appear to show that ESR may show slightly greater variability and therefore discriminative power in terms of detecting treatment effects. However plasma viscosity (PV) has now replaced ESR within the local region of this trial. Given limitations associated with the sensitivity and specificity of individual tests, both ESR and CRP tests will be conducted as proxies for inflammation within the proposed trial.

The possibility that magnet therapy may alleviate pain in chronic musculoskeletal conditions via endogenous opiod channels has also been considered. However the decision has been made not use blood samples for the purpose of exploring changes in endogenous opiods, such as beta-endorphin. This is due a number of factors, including the fact that endorphin tests are not routinely used within the NHS and therefore costs are likely to be prohibiting. Moreover, the requirement for additional blood samples involving non-routine tests is unlikely to be viewed as acceptable by research participants.

Additional disease specific outcome measures will consist of Patients’ Assessment of Physical Function and Subjects Global Assessment of Disease Activity (S-GADA) (Arnett et al 1988, Hochberg et al 1992, Felson et al 1993). Data provided by these instruments will be pooled together with other data to provide an overall index of disease activity (Pincus 2005).

Generic outcome measures will consist of the SF-36 and EuroQol. For the proposed trial these represent valid outcome measures in their own right and will also aid comparison of findings with other studies, such as MACROPOD. Participants will also be asked to report on their use of health care resources and personal expenditure relating to health care. This together with data collected using the EuroQol will facilitate any cost-effectiveness analysis of magnet therapy arising from this trail (EuroQol Group 1990).

Participants will be asked to report on compliance in terms of time spent wearing each device. This may related to treatment effectiveness and assurance will be required that participants actually wore the devices they were given. However poor compliance will not be used as a basis for exclusion from analyses. Success of blinding will be assessed by asking participants to judge whether or not each device worn was a placebo. Adverse events and reactions will be monitored throughout the trial.

**How will the outcome measures be measured at follow-up?**

**Questionnaire**

Questionnaires will be sent to participants two to three days before each planned follow up. Completed questionnaires will then collected by the chief investigator. These will contain all self-report outcome measures described previously.

**Pill Count**

Participants will be visited at home by the chief investigator at recruitment and at the end of each treatment phase. With the participants help and prior consent (obtained at recruitment) a ‘pill count’ will then be conducted. This will involve counting the remaining number of tablets or capsules that the participant has in their possession for each analgesic, NSAID or DMARD received on repeat prescription. Issue dates will also be recorded. At a later stage prescription records will be obtained from each participant’s general practice. Together this information will be used to estimate use of prescription medication during each treatment phase. This approach has been used successfully by both the RESPECT trial (Wong et al 2004) and the MACROPOD trial. Before this, participants will also be asked to estimate the extent to which their medication use altered during each phase, using a 5-point Likert scale. This questionnaire item will be used for the purpose of validating estimated medication use.

**Blood tests**

Participant consent will be required to provide blood samples on five separate occasions, i.e. shortly after recruitment and at each follow up. Blood samples will be taken by a phlebotomist, practice nurse or GP. Samples will be sent via normal NHS channels to local haematology laboratories for PV and CRP testing. Results will be entered into the patient’s medical records and collected for the trial at a later date. Once recruited, all participants will be followed up regardless of any failure to provide blood samples.

**Case Report Forms**

General practices will be encouraged to report all adverse events or reactions using standard forms provided. Similarly, participants will be asked to report any such events directly to the trial and inform their GP.

- 1. **What is the proposed sample size?**

The sample size for this trial was determined using *GPower* (Erdfelder et al). This suggested that complete data from 62 participants would provide 80% power to detect a minimal clinically important improvement of 20% in pain outcomes (Felson et al 1995) for the full strength device using a one way analysis of variance (P = 0.05). This assumed a mean score of 65 on a standard 100mm pain visual analogue scale (VAS) for the demagnetised device, with a difference of -13 for the full strength device and -6.5 for each of the placebo devices. An upper limit of 21.7mm was set for the common standard deviation within subjects. Estimates for both mean and standard deviation were based on data obtained by the MACROPOD trial, together with data presented by Segal and colleagues (2001).

Allowing for a maximum of 10% attrition from recruitment to final data collection, 69 participants will need to be recruited (refer to Section 3.12).

- 1. **What is the planned recruitment rate?**

216 potentially eligible patients with RA will be approached with information about the trial and invited to take part. Based on experience from the MACROPOD trial and taking into account the fact that blood samples will be required for participation, it is anticipated that roughly 50% (108) of patients will fail to respond.

For those who express an interest, a meeting will then be arranged. Patients will be sent an appointment letter together with a copy of the consent form and a baseline questionnaire. During the meeting the chief investigator will offer a detailed explanation of the trial, show examples of the devices involved and answer any questions. He will then assess eligibility, seek the patients’ written consent to participate in the trial and collect the baseline questionnaire. Meetings will normally take place within the patient’s home as a pill count will also be conducted if the person is recruited. At this stage it is estimated that up to 35% (38) patients will either be found to be ineligible or decline to participate. This should leave 69 patients who will then be entered into the trial.

Provided that an efficient method of arranging appointments is organised, either by means of secretarial support or by allowing approximately 4 weeks to field calls, then the actual period required for visiting and recruiting all participants should take no longer than 8 weeks in total. This assumes a figure of between 2 to 3 appointments per day, allowing for gaps in appointments and time spent on additional tasks, such as organising blood tests.

- 1. **Are there likely to be any problems with compliance?**

Non-compliance in terms of failure to wear devices for the recommended minimum of 12 hours per day is unlikely to represent a major problem. Compliance will be measured and systematic bias should be avoided due to the random allocation of participants to different intervention sequences. Whilst the present study employs placebo-control, the emphasis is nevertheless that of a pragmatic evaluation of the therapeutic effectiveness of magnet therapy within a real world setting (Schwartz and Lellouch 1967). Non-compliance therefore represents a valid outcome in its own right.

- 1. **What is the likely rate of loss to follow-up?**

It is anticipated that loss to follow-up should not exceed 10%. In relation to this figure it is worthwhile noting that just 1 participant out of 45 (2%) withdrew from the MACROPOD trial. However the duration for participation (16 weeks) was shorter than the proposed trial. More importantly, participants in the CAMBRA trial will be asked to provide blood samples on successive occasions. This may serve as a disincentive for continued participation. Efforts will be made however to continue follow up for participants who refuse to provide further blood samples.

- 1. **How many centres will be involved?**

Up to 12 general practices will be recruited in two phases. Initially the trial will seek to recruit nine large general practices over a period of one month from three primary care trusts: 1) Hull PCT; 2) East Riding of Yorkshire PCT; 3) Selby & York PCT. Practices approached will be those with four or more partners. This will help to ensure the availability of a practice nurse or phlebotomist at each site for the purpose of taking blood samples. Roughly equal numbers of patients with RA will then be contacted by each practice to help achieve a fairly representative sociodemographic cross section of this clinical population. Should the nine practices fail to identify a sufficiently large number of patients with RA, then an additional three practices will be recruited.

- 1. **Give details of the planned analyses**

Data from all participants recruited into the trial will be included in the analysis according to intention to treat. The basic statistical method used for the purpose of primary analysis will be that of analysis of variance. This will look for a significant difference in treatment outcomes amongst the four devices using follow up scores from each treatment phase. If magnet therapy of any true therapeutic benefit one would expect this to show a statistically significant difference between devices in favour of better outcomes for the experimental (full strength MagnaMax) device. However if a significant difference is observed with poorer outcomes for the demagnetised dummy device then the other three devices, then this would tend to indicate that any benefit of magnet therapy may be attributed simply to a placebo effect. This assumes that both attenuated and copper bracelets serve as valid placebos, which is an issue that will be explored in greater detail. Within this analysis a significant difference in treatment effects for the pain VAS (as the primary outcome measure) will be viewed as the critical test.

As a secondary step in the analysis, estimated treatment effects for each of the devices will also be adjusted according to medication use and compliance, for both of which continuous data will be available. The assumption here is that both of these variables are likely to modify the effects of treatment in terms of all major outcomes. Specifically it is believed that a reduction in either of these variables is likely to reduce the apparent effectiveness of magnet therapy. In pragmatic terms this may not be important, however for the majority of readers this may be interest. The basic approach used for this purpose will be that of multiple regression, testing first for non-linear relationships (Bland 2000).

Further refinement to analysis plan will be sought towards completion of the data collection stage of this trial. This will take into consideration advice from Professor Martin Bland and may involve adjusting for possible period effects as described using a ‘basic estimators approach’ outline in Section 5.4.1 of *Crossover Trials in Clinical Research 2ed* (Senn 2002)

Treatment effect estimates will be presented for each of the four devices together with their standard error and 95% confidence intervals. The computer package used for this purpose will be SPSS® and possibly also SAS®.

- 1. **Are there any planned subgroup analyses?**

Further analyses will be performed in order compare effects according to age, sex and primary location of pain. Differences in effect according to treatment preference will also be explored. The focus here will be on identifying possible baseline variables which may serve as useful predictors of treatment effectiveness, although dangers associated with multiple testing will be acknowledged. Similarly, this trial will explore a number of factors in terms of their possible role in mediating observed placebo effects. These will include level of education, belief in holistic and complementary medicine and belief in the paranormal. The latter of which will be measured at baseline using the HCAMQ (Hyland et al 2003) and Australian Sheep-Goat Scale (Thalbourne 2001).

- 1. **What is the proposed frequency of analyses?**

Analysis will be performed at the end of the trial only, although adverse events and reactions will be actively monitored.

- 1. **Will the trial address any economic issues?**

Quality of life, medication use and data relating to health care expenditure will be collected as part of the trial. If magnet therapy is shown to be effective for reducing either arthritis symptoms or medication use then a cost-effectiveness analysis will be conducted. This will be lead by staff from the Centre for Health Economics at The University of York.

- 1. **What is the estimated research cost for the trial?**

Total: £13,635 excluding salaries. This is based on: £6900 in order to reimburse general practices (see below); £2000 for research travel expenses; £1800 for device costs; £1035 for trial participant expenses; £1000 printing and administration; £500 focus group expenses; £400 telephone charges.

- 1. **What will be the NHS cost implications for the trial?**

The trial will reimburse general practices for costs incurred in supporting this trial. This relates to time of nurses, phlebotomists, clerical staff and GPs in performing research activities which place a strain on resources. Activities include identifying / contacting patients as part of recruitment. Clerical staff will also book and accommodate participant appointments. Blood samples will be taken from each participant on a maximum of four occasions. In each case this will involve two samples. In total, general practices will be reimbursed up to £100 per participant. Payments will be issued on the basis of £25 per visit. This assumes haematology charges of £10.15 for CRP, £6 for PV and £8.50 for staff time. This amounts to a maximum of £6900, although the actual figure is likely to be lower as some participants will fail to provide a complete set of blood samples.

- 1. **Over what period is funding requested?**

N/A

- 1. **Is a pilot feasibility stage planned?**

A pilot study has already been conducted in order to test several methodological features of the proposed trial, i.e. MACROPOD.

Before patient recruitment begins, two focus groups will be formed. One involving GPs, practice nurses and phlebotomists. Members of this group will be drawn from practices which express an interest in participating in CAMBRA. The other will consist of people with RA and those involved in providing direct support to people with RA. These will be drawn from members of community support groups for people with affected by RA and not via NHS channels.

Each focus group will be provided with a detailed outline for the proposed trial, together with trial materials in advance. Feedback will be sought on a variety of issues, including possible weaknesses with trial methodology and suitability of written material. The groups will help to identify any special considerations associated with the study of rheumatoid arthritis which may have been overlooked. Each group will also discuss issues specific to the use of blood tests, such as their acceptability to study volunteers. Information gathered will be used to refine the trial protocol. This may result in an application for the approval of minor amendments from local research ethics committee concerned.

1. **DETAILS OF THE TRIAL TEAM**
   1. **Trial management**

The Department of Health Sciences at The University of York will act as the co-ordinating centre for CAMBRA. This trial will be led by Stewart Richmond as the chief investigator, who will be responsible for clinical and scientific co-ordination, together with day to day management. This will include recruitment of all general practices and participants, data collection, analysis and dissemination of findings. Professor David Torgerson, as Director of the York Trials Unit will be responsible for supervising patient randomisation and data management. He will also act as an advisor on matters relating to trial design and methodology. Dr Hugh MacPherson will act advisor on matters concerning the use and evaluation of complementary and alternative medicine. Professor Martin Bland will serve as a statistical advisor to the trial. Professor Peter Campion from The University of Hull will advise on matter concerning general medical practice. As a co-investigator for MACROPOD, he will also contribute towards the evaluation of this trial. For the purpose of trial development further support will be sought from a leading rheumatologist.

- 1. **Participating centres**

Either nine or twelve general practices will be recruited across three PCT regions. Practice managers / practice staff will identify potentially eligible patients from medical records with support from the chief investigator. Honorary contracts will be obtained for the chief investigator for each of the PCTs involved. Administrative staff from each practice will then contact patients by post. Each patient will be sent an information sheet, letter of invite to participate and a standard covering letter expressing support for the trial, signed by one of the GPs. Patients will be asked to contact the chief investigator if they are interested in participation.

General practice will be informed when a patient is recruited into the trial. A series of four appointments will then be made for blood samples to be taken from the participant by either a nurse or a phlebotomist located within the practice. Administrative staff will be responsible for organising these appointments, sending samples for analysis and providing the trial with the results. Following all other data collection activities, each practice will be visited by the chief investigator during which prescription histories and blood tests results will be obtained.

1. **APPENDICES**
   1. **Appendix 1: Randomisation sequence for device allocation**

|  | | **Order of treatment phases (weeks)** | | | | | | | |
| --- | --- | --- | --- | --- | --- | --- | --- | --- | --- |
|  |  | **wk1** | **weeks**  **1 to 5** | **wk6** | **weeks**  **7 to 11** | **wk12** | **weeks**  **13 to 17** | **wk**  **18** | **weeks**  **19 to 23** |
| **Sequence Number** | **1** | Recruitment | *Full Strength* | wash out | *Attenuated* | wash out | *Demagnetised* | wash out | *Copper* |
|  | **2** |  | *Full Strength* |  | *Attenuated* |  | *Copper* |  | *Demagnetised* |
|  | **3** |  | *Full Strength* |  | *Demagnetised* |  | *Attenuated* |  | *Copper* |
|  | **4** |  | *Full Strength* |  | *Demagnetised* |  | *Copper* |  | *Attenuated* |
|  | **5** |  | *Full Strength* |  | *Copper* |  | *Attenuated* |  | *Demagnetised* |
|  | **6** |  | *Full Strength* |  | *Copper* |  | *Demagnetised* |  | *Attenuated* |
|  | **7** |  | *Attenuated* |  | *Full Strength* |  | *Demagnetised* |  | *Copper* |
|  | **8** |  | *Attenuated* |  | *Full Strength* |  | *Copper* |  | *Demagnetised* |
|  | **9** |  | *Attenuated* |  | *Demagnetised* |  | *Full Strength* |  | *Copper* |
|  | **10** |  | *Attenuated* |  | *Demagnetised* |  | *Copper* |  | *Full strength* |
|  | **11** |  | *Attenuated* |  | *Copper* |  | *Full Strength* |  | *Demagnetised* |
|  | **12** |  | *Attenuated* |  | *Copper* |  | *Demagnetised* |  | *Full Strength* |
|  | **13** |  | *Demagnetised* |  | *Full Strength* |  | *Attenuated* |  | *Copper* |
|  | **14** |  | *Demagnetised* |  | *Full Strength* |  | *Copper* |  | *Attenuated* |
|  | **15** |  | *Demagnetised* |  | *Attenuated* |  | *Full Strength* |  | *Copper* |
|  | **16** |  | *Demagnetised* |  | *Attenuated* |  | *Copper* |  | *Full strength* |
|  | **17** |  | *Demagnetised* |  | *Copper* |  | *Full Strength* |  | *Attenuated* |
|  | **18** |  | *Demagnetised* |  | *Copper* |  | *Attenuated* |  | *Full Strength* |
|  | **19** |  | *Copper* |  | *Full Strength* |  | *Attenuated* |  | *Demagnetised* |
|  | **20** |  | *Copper* |  | *Full Strength* |  | *Demagnetised* |  | *Attenuated* |
|  | **21** |  | *Copper* |  | *Attenuated* |  | *Full Strength* |  | *Demagnetised* |
|  | **22** |  | *Copper* |  | *Attenuated* |  | *Demagnetised* |  | *Full Strength* |
|  | **23** |  | *Copper* |  | *Demagnetised* |  | *Full Strength* |  | *Attenuated* |
|  | **24** |  | *Copper* |  | *Demagnetised* |  | *Attenuated* |  | *Full Strength* |

- 1. **Appendix 2: Participant flow – illustration of CONSORT diagram**

**Contact 216**

**50% fail to respond**

**Assess 108**

**35% decline or ineligible**

**Randomise 69 (24 possible sequences)**

**Treatment phase 1**

**Full data from 62**

**10% attrition**

**Treatment phase 2**

**Treatment phase 3**

**Treatment phase 4**

- 1. **Appendix 3: Complete trial timetable**

[*to be inserted*]

- 1. **Appendix 4: Recruitment schedule**

[*to be inserted*]

1. **REFERENCES**

Akobeng, A. (2005) Understanding randomised controlled trials. *Arch Dis Child*. 90, 840-844.

ARC (2000) *Complementary therapies and arthritis: an information booklet* (amended July 2005). Arthritis Research Campaign. Chesterfield.

ARC (2002) *Arthritis: The big picture*. Arthritis Research Campaign. Citing electronic resources: http://www.arc.org.uk/about_arth/bigpic.htm#6 (last accessed 08/01/05).

ARC (2003) *Rheumatoid arthritis: an information booklet*. Arthritis Research Campaign. Chesterfield.

Argoff, C. (2002) Pharmacological management of chronic pain. *JAOA.* 102(9) S21-S26.

Arnett, F., Edworthy, S., Bloch, D., McShane, D., Fries, J., et al (1988) The American Rheumatism Association 1987 revised criteria for the classification of rheumatoid arthritis. *Arthritis Rheum*. 31, 315-324.

Aspy, C., Carter, R., Hall, T., Mold, J. (2002). The effectiveness of magnet therapy for treatment of wrist pain attributed to carpal tunnel syndrome. *Journal of Family Practice* – January 2002. Citing electronic resources: http://www.jfponline.com/napcrg/display_one.asp?id=702 (last accessed 05/05/04).

Astin, J. A. (1998). Why patients use alternative medicine: results of a national survey. *JAMA.* 279, 1548-1553.

Avenell, A., Grant, A., McGee, M., McPherson, G., Campbell, M., et al (2004) The effects of an open design on trial participant recruitment, compliance and retention – a randomized controlled trial comparison with a blinded, placebo-controlled design. *Clinical Trials*. 1, 1-9.

Basford, J. R. (2001). A historical perspective of the popular use of electric and magnetic therapy. *Archives of Physical Medicine and Rehabilitation*. 82, 1261-1269.

Bellamy, N., Campbell, J., Syrotuik, J. (1999) Comparative study of self-rating pain scales in rheumatoid arthritis. *Curr Med Res Opin*. 15(2), 121-127.

Bland, M. (2000) *An introduction to medical statistics* (3^rd^ ed). Oxford. Oxford University Press.

Campbell, A. (2003). Placebo: the belief effect. *Complementary Therapies in Medicine.* 11, 125-128.

Department of Health (2001) *Government response to the House of Lords Select Committee on Science and Technology’s report on complementary and alternative medicines*. CM 5124, HMSO. Citing electronic resources: http://www.archive.official-documents.co.uk/document/cm51/5124/5124.pdf (last accessed 05/05/04).

Duncan, G. (1989). Comparison of verbal and visual analogue scales for measuring the intensity and unpleasantness of experimental pain. *Pain*. 57, 295-303.

Eccles, N. (2005) A critical review of randomized controlled trials of static magnets for pain relief. *Journal of Alternative and Complementary Medicine*. 11(3), 495-509.

Emery, P. (2006) Treatment of rheumatoid arthritis. *BMJ*. 332, 152-155.

Erdfelder E, Faul F, Buchner, A (1996). GPOWER: A general power analysis program. Behavior Research Methods, Instruments, & Computers, 28, 1-11.

Ernst, E. (2004) Musculoskeletal conditions and complementary / alternative medicine. *Best Practice & Research: Clinical Rheumatology*. 18(4), 539-556.

EuroQol Group (1990) EuroQol – a new facility for the measurement of health related quality of life. *Health Policy*. 16, 199-208.

Felson, D., Anderson, J., Boers, M., Bombadier, C., Chernoff, M., et al (1993) The American College of Rheumatology preliminary core set of disease activity measures for rheumatoid arthritis clinical trials. *Arthritis Rheum.* 36, 729-740.

Felson, D., Anderson, J., Boers, M., Bombardier, C., Furst, D., et al (1995) ACR preliminary definition of improvement in rheumatoid arthritis. *Arthritis & Rheumatism*. 38(6)

Fergusson, D., Glass, K., Waring, D., Shapiro, S. (2004) Turning a blind eye: the success of blinding reported in a random sample of randomised, placebo controlled trials. *BMJ*. 328, 432-437.

Finegold, L. and Flamm, B. (2006) Magnet therapy: extraordinary claims, but no proved benefits. *BMJ*. 332, 4.

Grennan, D., Knudson, J., Dunckley, J., et al. (1980). Serum copper and zinc in rheumatoid arthritis and osteoarthritis. *New Zealand Medical Journal.* Vol 91, issue 652, pp 47-50.

Harlow, T., Greaves, C., White, A., Brown, L., Hart, E., et al. (2004). Randomised controlled trial of magnetic bracelets for relieving pain in osteoarthritis of the hip and knee. *BMJ.* 329, 1450-1454.

Hinman, M. (2002) The therapeutic use of magnets: a review of recent research. *Phys Ther Rev*. 7(1), 33-43.

Hochberg, M., Chang, R., Dwosh, I., Lindsey, S., Pincus, T., et al (1992) The American College of Rheumatology 1991 revised criteria for the classification of global functional status in rheumatoid arthritis. *Arthritis Rheum*. 35, 498-502.

House of Lords Select Committee on Science and Technology. *Complementary and alternative medicine, 6^th^ report*. Session 1999-2000, HL Paper 123., HMSO, 2000. Citing electronic resources: http://www.parliament.the-stationery-office.co.uk/pa/ld199900/ldselect/ldsctech/123/12302.htm#a1 (last accessed 05/05/04).

Hyland, M., Lewith, G., Westoby, C. (2003). Developing a measure of attitudes: the holistic complementary and alternative medicine questionnaire. *Complementary Therapies in Medicine*. 11, 33-37.

Jordan, K., Sawyer, S., Coakley, P., Smith, H., Cooper, C., et al (2004). The use of conventional and complementary treatments for knee osteoarthritis in the community. *Rheumatology (Oxford)*. 43, 381-384.

Madhok, R., Kerr, H., Capell, H. (2000) Recent advances: rheumatology. *BMJ*. 321, 882-885.

Malaise, M., Franchimont, P. (1987) Methods of clinical and biological assessment of rheumatoid arthritis. *Scand J Rheumatol Suppl.* 65, 81-84.

Milanino, R., Frigo, A., Bambara, L., Marrella, M., Moretti, U., Pasqualicchio, M., Biasi, D., Gasperini, R., Mainenti, L., Velo, G. (1993). Copper and zinc status in rheumatoid arthritis: studies of plasma, erythrocytes, and urine, and their relationship to disease activity markers and pharmacological treatment. *Clinical and Experimental Rheumatology*. 11(3), 271-281.

NHSE (1998). *In the Public Interest, Developing a Strategy for Public Participation in the NHS*. Department of Health.

Patterson, C. and Dieppe, P. (2005) Characteristics and incidental (placebo) effects in complex interventions such as acupuncture. *BMJ*. 330, 1202-1205.

Pincus, T. (2005) The American College of Rheumatology (ACR) Core Data Set and derivative "patient only" indices to assess rheumatoid arthritis. *Clin Exp Rheumatol*. 23(5 Suppl 39), S109-S113.

Pope, K. and McNally, R. (2002). Nonspecific placebo effects explain the therapeutic benefit of magnets. *Scientific Review of Alternative Medicine*. Winter, Vo 7.

Ratterman, R., Secrest, J., Norwood, B., Chi’ien, A. (2002) Magnet therapy: what’s the attraction? *J Am Acad Nurse Pract*. 14(8), 374-353.

Rindfleisch, J., Muller, D. (2005) Diagnosis and management of rheumatoid arthritis. *American Family Physician*. 72(6), 1037-1047.

Rodriguez, L. and Jick, H. (1994). Risk of upper gastrointestinal bleeding and perforation associated with individual non-steroidal anti-inflammatory drugs. *The Lancet*. 343, 769-772.

Schwartz, D., Lellouch, J. (1967) Explanatory and pragmatic attitudes in therapeutic trials. *J. chron. Dis.* 20, 637-648.

Segal, N., Toda, Y., Huston, J., Saeki, Y., Shimizu, F., et al. (2001). Two configurations of static magnetic fields for treating rheumatoid arthritis of the knee: a double-blind clinical trial. *Archives of Physical Medicine and Rehabilitation*. Oct, p.82.

Senn, S. (2002) *Crossover trials in clinical research*. Chichester. John Wiley & Sons Ltd.

Schulz, K. and Grimes, D. (2002) Blinding in randomised trials: hiding who got what. *The Lancet*. 359, 696-700.

Shackel, N., O Day, R., Kellet, B., Brooks, P. (1997). Copper-salicylate gel for pain relief in osteoarthritis: a randomised controlled trial. *Medical Journal of Australia*. 167(3), 134-136.

Thalbourne, M. (2001) Measures of the sheep-goat variable, transliminality, and their correlates. *Psychol Rep*. 88(2), 339-350.

Thomas, K., Nicholl, J., Coleman, P. (2001). Use and expenditure on complementary medicine in England: a population based survey. *Complementary Therapies in Medicine.* 9, 2-11.

Trock, D (2000) Electromagnetic fields and magnets: investigational treatment for musculoskeletal disorders. *Rheumatic Disease Clinics of North America*. 26(1), 51-62.

Vickers, A. and de Craen, A. (2000) Why use placebos in clinical trials? A narrative review of the methodological literature. *Journal of Clinical Epidemiology*. 53, 157–161.

Walker-Bone, K., Javaid, K., Arden, N., Cooper, C. (2000) Regular review: Medical management of osteoarthritis. *BMJ*. 321, 936-940.

Walker, W., Keats, D. (1976). An investigation of the therapeutic value of the ‘copper bracelet’ – dermal assimilation of copper in arthritic/rheumatoid conditions. *Agents and Actions*. 6(4), 454-459.

Walker, W., Beveridge, S., Whitehouse, M. (1981) Dermal copper drugs: the copper bracelet and Cu(II) salicylate complexes. *Agents and Actions Supplements*. 8, 359-367.

Weber, C. (1984) *Copper response to rheumatoid arthritis*. Med Hypotheses. 15(4), 333-348.

Weintraub, I. (1999) Magnetic bio-stimulation in painful diabetic peripheral neuropathy: a novel intervention – a randomised, double-blind crossover study. *American Journal of Pain Management*. 9(1), 8-17.

Wider, B., Ernst, E. (2003) CAM research funding in the UK: survey of medical charities in 1999 and 2002. *Complementary Therapies in Medicine*. 11, 165-167.

Wong, I., Campion, P., Coulton, S., Cross, B., Edmondson, H., et al (2004). Pharmaceutical care for elderly patients shared between community pharmacists and general practitioners: a randomised evaluation (RESPECT). *BMC Health Services Research*. 4(11). Citing electronic resources: http://www.biomedcentral.com/1472-6963/4/11 (last accessed June 2004).

Youssef, A., Wood, B., Baron, D. (1983). Serum copper: A marker of disease activity in rheumatoid arthritis. *Journal of Clinical Pathology.* 36(1), 14-17.
